# Treatment of unstable distal radius fractures: non-invasive dynamic external fixator versus volar locking plate – functional and radiological outcome in a prospective case-controlled series

**DOI:** 10.1051/sicotj/2015033

**Published:** 2015-12-16

**Authors:** Ali S. Bajwa, Manju Rammappa, Ling Lee, Rajesh Nanda

**Affiliations:** 1 Villar Bajwa Practice (London and Cambridge) 30 Devonshire Street London W1G 6PU UK; 2 James Cook University Hospital Marton Road Middlesbrough TS43BW UK; 3 University Hospital of North Durham North Road Durham DH15TW UK; 4 University Hospital of North Tees Hardwick Road TS198PE Stockton-on-Tees UK

**Keywords:** Distal radius fracture, volar locking plate, external fixture, Functional result, Non-invasive fixator

## Abstract

*Introduction*: Distal radius fracture (DRF) is a common injury and various treatment modalities including open reduction and internal fixation (ORIF) with volar locking plate are available. More recently, a non-invasive external fixator has been used.

*Aims*: To prospectively compare the use of a non-invasive external fixator with early dynamisation for DRF against ORIF with volar locking plate control group.

*Methods*: Consecutive patients with closed DRF were included in a prospective case-controlled study. Patients were assigned to non-invasive external fixator or ORIF. Minimum follow-up was two years. Follow-up was at weeks 2, 4, 6, 8, 12, 26 and at one and two-year post-operatively. The outcome measures included demographic details, injury mechanism, AO fracture type, risk factors, body mass index (BMI), ulnar styloid fracture and dorsal comminution, radiographs, grip strength and DASH score.

*Results*: Consecutive 50 patients were treated either with non-invasive external fixator (25/50) or with ORIF (25/50) and the mean age of the two groups was 53 years (*SD* 17.1) and 49 years (*SD* 19.5), respectively. Demographics were matched in two groups. In the non-invasive external fixator group, there were 10 AO Type-A, 5 Type-B and 10 Type-C fractures. The ORIF group included 8 Type-A, 6 Type-B and 11 Type-C fractures. The mean DASH score at three-months and one-year post-injury in non-invasive fixator group was 12.2 (*SD* 3.1) and 3.5 (*SD* 0.7), respectively, significantly greater than those of ORIF group 14.5 (*SD* 5.6) and 11.2 (*SD* 4.4), respectively (*p* < 0.05).

*Conclusion*: DRF treated with non-invasive external fixator can give functional results superior to ORIF at three-months and the trend is maintained at one and two-year post-operatively.

## Introduction

Distal radius fracture (DRF) is a common injury with bimodal distribution including the high-energy injuries in young population and a second rise in incidence in older population with osteoporosis [1]. DRF with dorsal angulation is by far the commonest pattern and is often described as the Colles’ fracture [2]. The principal goal of fracture treatment in DRF is not only to achieve bony union but also to have a pain-free and well-functioning limb [3]. This can be accomplished with different surgical approaches, some being more invasive than others. In general, it is preferred and cost-effective if the optimal function is achieved without recourse to invasive surgery [4].

Recently concluded UK DRAFFT study showed no significant advantage of open reduction and internal fixation (ORIF) with volar locking plate over closed reduction and stabilisation with Kirschner (K) wires with plaster cast application [5]. ORCHID study group reported marginally superior results with fixed angle volar plates in DRF over conservative measures, however the improvement did not achieve statistical significance in 149 patients [6]. DRF is the second most common adult fracture and a considerable burden on health economy [1] while patients are seeking, and rightly so, better functional outcomes. However, in general there is no consensus on treatment of distal radius fracture [3] and various modalities are available including ORIF with volar or dorsal plate osteosynthesis, external fixation in bridging or non-bridging modes and immobilisation in casts with or without K-wires [7, 8]. There are inherent advantages and disadvantages with each approach of DRF treatment. In summary, ORIF has the potential risk of infection, neurovascular injury, tendon irritation or rupture, carpal tunnel syndrome and being more resource intensive [9], while the invasive external fixation has increased risk of pin site infection, stiffness and neurological injury [9]. The non-invasive or Kirschner wire (K-wire) use may lead to poor reduction, late collapse of the fracture after removal of wires and stiffness due to several weeks of joint immobilisation [10]. More recently, non-invasive external fixator with the option of dynamisation has been used successfully in treating DRF. We prospectively audited the use of non-invasive and minimally invasive approach to treating distal radius fractures with a recently regulatory approved non-invasive external fixator, which allows dynamisation of the wrist during fracture treatment. The aim of the study was to prospectively audit the functional outcome in patients using this approach and comparing it with the use of ORIF with volar locking plate group.

## Methods

**Figure 1.**
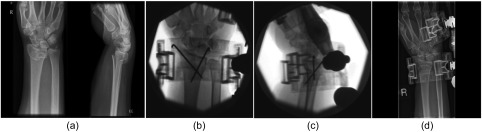
Radiographs and fluoroscopic images of a 35-year-old female patient with unstable intra-articular DRF (a) treated with non-invasive dynamic external fixator after closed reduction and percutaneous K-wire fixation (b and c). She had wires removed at three weeks post-op with continuation of non-invasive external fixator in dynamic mode (d) for further three weeks.

**Figure 2.**
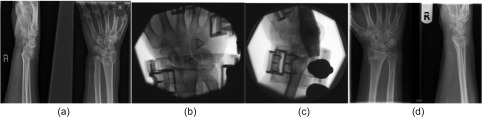
Radiographs and fluoroscopic images of a 65-year-old patient with unstable extra-articular DRF (a) treated with non-invasive dynamic external fixator (b and c). There was no collapse or shortening at the fracture at six-month post-op radiographs (d).

**Figure 3.**
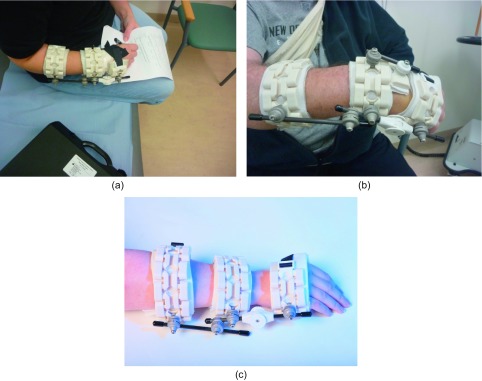
(a) Non-invasive fixator (*Cambfix*, *Polyarmour*) on the right forearm of a female patient with DRF who can hold a pen, mobilise the wrist and rest the forearm. (b) Non-invasive fixator (*Cambfix*, *Polyarmour*) on a male patient in standard configuration: three articulated radiolucent bracelets lined with waterproof polylattice membrane, interconnected by carbon fibre rods, which are captured by connectors that allow 360 degree freedom of movement before setting the system rigid. (c) Cambfix device on the wrist showing three articulated bracelets with interconnecting rods and selectively lockable hinge on the ulnar side. (Photograph courtesy of Cambfix Ltd).

The design was that of a case control study in a prospective cohort of patients with DRF that was followed up for two years and the results were audited. The aim of the study was to compare DRF treated with a non-invasive external fixation device and compare it with a control group using ORIF with volar locking plate. The DRF was defined as the fracture within 3 cm of the radiocarpal joint. The inclusion criteria were adult patients (>18 years) who had the mental ability to give informed consent for treatment, closed fracture or minimally open (Gustillo Grade 1) with fractures presenting within two weeks of sustaining injury. The exclusion criteria were the presence of neurovascular compromise, fracture more proximal than 3 cm from the radiocarpal joint or contraindication to anaesthetic. On review in the fracture clinic, the decision was made by the treating surgeon whether further surgical intervention was required based on patient factors, fracture reduction and stability [7]. The patients were assessed for the inclusion criteria for application of non-invasive external fixator with or without K-wires, or application of volar locking plate with open reduction and internal fixation. The process was non-randomised. The application was supplemented with K-wires after dynamic assessment of the fracture (Figure 1). If the fracture was unstable only in dorsal plane it was treated by non-invasive external fixator alone (Figure 2), however, the fractures that were unstable in more than one plane or predisposition to collapse on dynamic testing under image intensifier or with intra-articular extension received additional 1.6 mm K-wires, which were used in a standard configuration of either one or two dorsal and one radial wire inserted percutaneously in the safe corridors around the wrist (Figure 1). The procedure was carried as a day-case and the patients were allowed home after monitoring the distal neurovascular status. The non-invasive external fixator (*Cambfix*, *Polyarmour*, *Newcastle-upon-Tyne*, *UK*) used in this group of DRF patients comprises of three articulated bracelets (rings), which can conform to varying sizes of limbs using built in articulations and can be made rigid once optimal conformity is achieved. The polyurethane rings are interconnected using carbon fibre rods with adjustable connectors (Figures 1–3). The device was always used in the bridging mode with the presence of hinge at the level of mid-carpal joint to allow for early mobilisation of the wrist. This dynamisation using the selective hinge was commenced at two weeks after fixation. The radiographs were taken without removing the fixator (Figure 1). In the cohort of patients where ORIF with application of volar locking plate the surgery was carried out by a consultant grade surgeon or under their direct supervision. The procedure was conducted under intravenous antibiotics and tourniquet control using a volar approach. Volar locking plates used were either LCP Distal radius system (*Synthes*, *Switzerland*) or Medartis distal radius 2.5 (*Medartis*, *Basel*, *Switzerland*).

The follow-up was at 2, 4, 6, 8, 12 and 26 weeks clinically, radiologically and with patient reported outcome measures and then at 12 and 24 months with patient reported questionnaires. The outcome measures included functional score using validated DASH (disabilities of arm, shoulder and hand) score and radiographic assessment with antero-posterior (AP) and lateral radiographs. Grip strength was expressed as a percentage of the non-injured hand using a hand dynamometer and the range of motion was described as a percentage of the contralateral uninjured wrist. Patient demographic details, co-morbidities and AO fracture type were also documented. Risk factors that may lead to loss of reduction were noted including age > 60, osteoporosis, ulnar styloid fracture and dorsal comminution. The data were collected prospectively and analysed using Excel (*Microsoft*, *Redmond*, *Washington*). Statistical analysis included the descriptive statistics for nominal data, *t*-test for parametric data and chi-squared test for non-parametric data. For statistical testing the power of the study was set at 80% and significance value (*p*) at 5%.

## Results

Over a period of one year, 50 consecutive patients who had indications for surgical intervention and had either a non-invasive external fixator (*n* = 25) or an ORIF with a volar locking plate (*n* = 25). All patients had preliminary treatment either at the local accident and emergency department or outside the region. Patients with adequate reduction and stable configuration were left in plaster casts and were excluded. Following clinical and radiological review, 50 patients were judged to be candidates for further surgical intervention owing to poor fracture reduction and stabilisation. A total of 25/50 patients underwent application of a non-invasive external fixator (*Cambfix*, *Polyarmour*, *Newcastle-upon-Tyne*, *UK*). In the same period another 25 consecutive patients that underwent ORIF with volar locking plate for DRF were included as a control group. Data in two groups were matched for age and gender. The cohorts of patients were prospectively followed up. The demographic details are outlined in Table 1.

**Table 1.** Patient demographics in non-invasive fixator group.

Age	Mean 53 years (*SD* 17.1, range 19–82)
Gender	Females 16/25 (64%)
	Males 9/25 (36%)
Handedness	Right 15/25 (60%)
	Left 10/25 (40%)
Dominant hand injury	21/25 (84%)
BMI	Mean 32.1 (*SD* 3.7, range 24–41)

In the non-invasive fixator group the majority of patients (21/25) sustained an injury to their dominant hand. The mean age was 53 years (*SD* 17.1), however, there was a wide range (Table 1). The mean body mass index (BMI) was 32.5 (*SD* 3.7, range 24–41). This was not significantly different to the ORIF group (Table 2). The mechanisms of injury in non-invasive fixator group included falling from a standing height (16/25), mountain bike fall (2/25), road traffic accident (2/25), roller skates injury (1/25), injury on a sports field (1/25), fall from a sitting height (1/25), horse riding accident (1/25) and skiing injury (1/25). In comparison with the ORIF group the mechanism of injury included fall from standing height (18/25), biking injury (3/25), road traffic accident (2/25), injury on sports field (1/25) and fall from ladder (1/25). The majority of patients the sustained injury was a fall from standing height (34/50). The mean time from injury to presentation in the orthopaedic trauma unit was 3.8 days (*SD* 3.1, range 1–13) and the mean time from presentation to definitive treatment was 1.2 days (*SD* 0.6, range 0–3). In all cases patients had preliminary treatment and assessment by the accident and emergency unit (21/25) or by the orthopaedic team (4/25). On fracture clinic review, the decision was made by the treating surgeon whether further surgical intervention was required. If the patient fulfilled the inclusion criteria for the study, then a choice was made to treat with the application of a non-invasive external fixator with/without K-wires or the choice was made to treat with ORIF and volar locking plate. In the cohort of 25 patients where a non-invasive external fixator was used, there were 10 AO Type-A, 5 Type-B and 10 Type-C fractures. Among the Type-A fractures, two were A-2 and eight were A-3 subtypes. The Type-B group included one B-1 and four B-2 fractures while the Type-C group had six C-1 and four C-2 fractures. The ORIF group included 8 Type-A (two A-2, six A-3), 6 Type-B (one B-1, five B-2), and 11 Type-C (five C-1, five C-2, one C-3). All patients in the non-invasive fixator group were discharged from the ward as day-case patients and were advised on finger movements and elevation of the hand while patients in the ORIF group were mostly treated as inpatients (21/25). The initial clinical review was carried out at two weeks post-operatively and AP and lateral radiographs of the wrist were taken. In the non-invasive fixator group, the dynamisation in the selective range of motion was commenced using the hinge at the mid-carpal level. In this group K-wires were used in 15/25 patients and were removed at the four-week review except in three cases, which had returned to clinic at three weeks for a review because of clinic logistics and hence wires were removed at the same time and dynamisation of the fixator was commenced (Figure 1).

**Table 2.** Patient demographics in volar locking plate group.

Age	Mean 49 years (*SD* 19.5, range 21–74)
Gender	Females 14/25 (56%)
	Males 11/25 (44%)
Handedness	Right 17/25 (68%)
	Left 8/25 (32%)
Dominant hand injury	23/25 (92%)
BMI	Mean 31.5 (*SD* 4.3, range 22–39)

In the non-invasive fixator group, mean time to fracture union based on visible callus in two radiographic views was 4.4 weeks (*SD* 1, range 4–6) while the fixator application on the wrist was maintained on the wrist for a mean of 7.0 weeks (*SD* 1.2, range 4.7–8.1). The ORIF group showed fracture healing (callus formation in two views) at a mean of 7.8 weeks (*SD* 3.2, range 6–12). However, the ORIF group underwent fixation with a rigid locking plate, which may have prevented the formation of callus. At the end of the fracture treatment when the fixator was removed, all but one of the patients had already regained near normal range of motion (ROM) within 92% of the ROM in the uninjured wrist. In the ORIF group at six-week mark, only 16/25 (64%) patients achieved near normal ROM within 92% of contralateral wrist. One patient required physiotherapy rehabitation for four weeks before full functional recovery was noted in the non-invasive fixator group while in the control group (ORIF) 9/25 patients required four weeks or longer duration of physiotherapy. The grip strength was documented at a mean of 84% of the opposite hand (*SD* 7.8, range 68–96) at the time of removal of the fixator in the non-invasive fixator group, which was comparable to the control (ORIF) group at a mean of 82% of the contralateral hand (*SD* 6.9, range 45–95). The principal outcome measure was the DASH score, which improved at three months post-injury to a mean of 12.2 (*SD* 3.1, range 6–18) and 14.5 (*SD* 5.6, range 7–22) in non-invasive fixator group and control (ORIF) group, respectively. The improvement was greater in the non-invasive group (*p* < 0.05). In the non-invasive fixator group the DASH score further improved to a mean of 3.5 (*SD* 0.7, range 1–6) with 25/5 patients responding at one-year. In the control group (ORIF), the mean DASH score at one-year post-op was 11.2 (SD 4.4, range 1–19) with 24/25 patients responding. The improvement was sustained at a two-year follow-up in both groups but there was no significant improvement after one-year at which stage a total of 49/50 patients responded.

In the non-invasive external fixator group, there was no significant difference in mean DASH score at three-month follow-up in 15 patients with intra-articular fractures and those with extra-articular DRF (*t*-test, *p* = 0.78). Mean grip strength as percentage of the contralateral hand showed no significant difference between intra and extra-articular fracture at three-month follow-up (*t*-test, *p* = 0.88). There was significant difference in improvement in mean DASH score from 12.2 at three-month post-op to 3.5 at one-year follow-up (*t*-test, *p* < 0.05). In the non-invasive fixator group, the mean BMI of the patients treated was 32.5 (*SD* 3.7) and 16 patients had a BMI > 30, however no significant difference was noted in the mean DASH score compared to those with BMI 30 or less (χ^2^, *p* > 0.05). No return to operating theatre was needed in any of the patients during the acute treatment of the fracture or during the subsequent two years for a problem related to the wrist in the non-invasive fixator group. One patient required adjustment of the forearm bracelet while being monitored in the ward and one patient required physiotherapy rehabilitation. Fracture union was achieved in all patients and at 12 weeks post-injury radiographs dorsal collapse was not noted in any of the patients.

In the control (ORIF) group, one patient returned to theatre for removal of metal work due to irritation of extensor tendons by the tips of locking screws. There were 2/25 cases of superficial wound infection that settled with antibiotic therapy and further two cases (2/25) of stiffness that appeared to be related to complex regional pain syndrome (CRPS) and one case reported late onset (six-month post-op) of carpal tunnel syndrome, which was managed non-operatively.

No significant radiological collapse was noticed in either group. The radiological collapse was defined as a dorsal collapse beyond neutral in a lateral radiograph, radial length shortening by >3 mm in AP radiograph or articular step off >1 mm in either plane.

In the non-invasive fixator group, the median patient satisfaction score as recorded on a visual analogue score (VAS) at 8 weeks post-injury was 8, which improved to 9 at 12 weeks and was 10 at one-year and two-year follow-up (mean 9.2, *SD* 1.8, range 7–10). In the control group, high levels of satisfaction were seen except in two cases of CRPS who had a low VAS for satisfaction (4 and 5). Overall the median VAS for the control group (ORIF) was seven at 8 weeks post-operatively, which improved to nine at the one-year mark (mean 8.6, *SD* 4.2, range 4–10).

## Discussion

The DRF treatment is controversial owing to a number of fracture fixation choices and lack of consensus [3]. The recently published UK DRAFFT study has highlighted the fact that DRF is being overtreated by ORIF with the volar locking plate, which exposes patients to potentially higher surgical risks without achieving a significantly better outcome compared with non-invasive and minimally invasive measures [5]. The problems inherent in conventional non-operative treatment such as plaster cast include radiological evidence of collapse at the fracture site once wires or casts are removed and a functional deficit [10]. The immobilisation of the wrist leads to wasting of the muscles, reduced grip strength and stiffness of the wrist joint. The functional parameters improve gradually but do not always return to normality [10]. DRF treated with the *Cambfix Polyarmour* non-invasive external fixator showed a promising pattern of functional results in this prospective cohort. The DASH score at three-month follow-up was superior to patients treated with ORIF and plate osteosynthesis (*p* < 0.05). The DASH score improved at three months post-injury to a mean of 12.2 (*SD* 3.1, range 6–18) and 14.5 (*SD* 5.6, range 7–22) in the non-invasive fixator group and control (ORIF) group, respectively. At the one-year mark the DASH score in the non-invasive fixator group further improved to 3.5 (*SD* 0.7, range 1–6) and in the control group (ORIF) to 11.2 (*SD* 4.4, range 1–19). The improvement in the non-invasive fixator group remained superior to that in the ORIF group. This is despite the fact that the ORIF group had results comparable to those noted in the literature. Sügün et al. [11] reported a mean DASH score of 15.9 (range 0–72) after volar locking plate for DRF while Knight et al. reported more cautious results when ORIF of unstable DRF that resulted in a DASH score of 23 with intra-articular penetration of screws subsequent to fracture collapse was seen in 11/40 cases [12].

The improvement in DASH score at three months is remarkable but at one-year it improves further, emphasising a consistent improvement in the long-term results. This improvement was sustained at two-year follow-up as well. However, it is to be noted that one patient was a non-responder in the volar locking group at one and two year stage. Although the DASH score of this 51-year old patient (BMI 31) was reported to be 11 and 9 at both the three and six-month follow-ups respectively. It is however, difficult to be certain that a late deterioration did not occur in this particular patient. The results indicate no difference in mean DASH scores and grip strength of intra and extra-articular fractures, however the authors note that the sample size is relatively small and can lead to a type-2 error. Similarly, the number of patients with BMI 30 or less is small (*n* = 11) and hence statistical comparison is difficult.

The early functional recovery observed in this cohort appears to be reflected in the fact that dynamic movement at the wrist is commenced at two weeks post-operatively. This movement was initially commenced selectively in the volar direction in a dorsally unstable fracture and gradually incorporated free activity over six weeks at the selectively lockable hinge, which was positioned at the mid-carpal level on the ulnar side of the wrist (Figure 3a). The position of the hinge on the ulnar side avoided interference with the first carpometacarpal (CMC) joint movement and movement of the thumb. The hinge being on the ulnar side and mounted on a non-invasive device avoids the need for shifting the axis of rotation dorsally, which may have compromised results in other systems in the past since the bridging external fixator often uses invasive pins mounted on a metacarpal and the distal radius and cannot achieve placement of a hinge parallel to the axis of rotation of the joint. The early dynamisation has the potential to cause micro-movement at the fracture site, which may have contributed to rapid fracture healing at a mean of 4.4 weeks [13]. The assessment of fracture healing though is notoriously difficult and depends on the subjective element in clinical examination, the adequacy of radiographs and the exact timing of review. However, the fracture healing was confirmed in this series by confirming the absence of significant clinical tenderness at the fracture site and radiological evidence of callus formation in at least two planes. The use of a more sensitive radiological investigation such as CT or MRI scan was both beyond the clinical need and the available resource. In the non-invasive fixator group, the fracture fixation device was kept in situ for longer than traditional casts to ensure avoidance of delayed collapse often seen with removal of plaster casts in unstable fractures. The hinge was left unlocked during the final phase of healing to avoid stiffness in the wrist joint.

The patient satisfaction score on VAS was an important aspect of the study since the traditional invasive external fixators and external casts are poorly tolerated by some patients. Overall, the patients in this cohort had a good level of satisfaction with the comfort level. It is postulated that the double-lattice polymembrane impregnated with silicone offered an optimal level of comfort next to the skin and the lightweight glass-filled polyurethane and carbon fibre materials offered a lightweight construct thus allowing the patients to manage day-to-day activities with relative ease (Figure 3b).

There were two minor complications during the treatment and follow-up period. One patient, after the application of the non-invasive external fixator (Cambfix Polyarmour) with K-wires complained of discomfort around the forearm while being observed on the day-case unit and needed adjustment of the forearm bracelet, which was impinging on the skin. The patient was able to safely go home a few hours later on the same day. Another patient with an AO B-2 fracture who was treated with the non-invasive external fixator and K-wires had a reduced range of motion despite early mobilisation and required four weeks of physiotherapy to regain near normal (within 90% of the contralateral side) range of motion.

In 15/25 patients where adjunctive K-wire fixation was used, no difference was observed in functional outcome compared to 10/25 patients without K-wires. The patients treated with the addition of K-wires had an intra-articular extension of the fracture. The K-wires were removed at four weeks in 12/15 cases and in three patients the removal was carried out at three weeks post-operatively. No significant radiological collapse was noticed in either group. The radiological collapse was defined as a dorsal collapse beyond neutral in a lateral radiograph, radial length shortening by >3 mm in AP radiograph or articular step off >1 mm in either plane. The success of avoiding radiological collapse is partly in the selective use of K-wires in unstable fractures and the use of the device for a longer period than simply keeping it in situ until the radiological union occurs. In addition, the pressure pad was used over the unstable fracture fragment using rod-cuff-pad connectors (Figure 3b). The pressure pad allows for additional stability in addition to the potential three-point fixation and potential hydrostatic stability that the device has been designed to provide as the principal means of fracture fixation.

There have been reports in the literature of an increase in the use of ORIF and plate osteosynthesis by Yoon and Grewal, 2012 [14] during the past decade, without robust evidence of it being superior to non-invasive or minimally invasive treatment of DRF [9]. There are also reported complications of volar and dorsal locking plates [15, 12]. The innovative approach to combine the benefits of non-invasive treatment, avoidance of soft tissue stripping associated with invasive surgery and allowing early range of motion, appears to give satisfactory results in this prospective series. This approach relies on an understanding of the anatomical and biomechanical characteristics of individual DRF and tailoring the procedure and rehabilitation plan accordingly [7, 16].

## Conclusions

DRFs treated with a non-invasive external fixator (*Cambfix*, *Polyarmour*, *Newcastle-upon-Tyne*) can give functional results superior to ORIF at three-months and the trend is maintained at one and two-year post-operatively. The majority of closed DRFs can be treated using the non-invasive external fixator (*Cambfix*, *Polyarmour*, *Newcastle-upon-Tyne*) employing a completely non-invasive or minimally invasive approach and hence avoiding the need for open surgery provided the fracture can reduced closed. The functional outcome is superior to matched ORIF group while radiological parameters were well maintained in both groups. Functional results continue to improve for one-year in both groups and then plateau off. The use of non-invasive fixator is recommended in DRFs where closed reduction is possible. Further research in this key area, which is often neglected, is encouraged.

## Conflict of interest

Dr. Bajwa reports being part of design team for Non-invasive external fixator (Cambfix Ltd). He is a stock holder in Cambfix Ltd, outside the submitted work; In addition, Dr. Bajwa has a patents issued and pending.

None of the other authors have any relevant disclosures.
